# Combined Oral Contraceptive Treatment Does Not Alter the Gut Microbiome but Affects Amino Acid Metabolism in Sera of Obese Girls With Polycystic Ovary Syndrome

**DOI:** 10.3389/fphys.2022.887077

**Published:** 2022-06-21

**Authors:** Beza Tayachew, Heidi Vanden Brink, Yesenia Garcia-Reyes, Haseeb Rahat, Angelo D'Alessandro, Daniel N. Frank, Charles E. Robertson, Lori Silveira, Megan Kelsey, Laura Pyle, Melanie Cree-Green

**Affiliations:** ^1^ Division of Pediatric Endocrinology, University of Colorado Anschutz Medical Campus, Aurora, CO, United States; ^2^ Department of Medicine, Division of Infectious Diseases, University of Colorado Anschutz Medical Campus, Aurora, CO, United States; ^3^ Division of Nutritional Sciences, Cornell University, Ithaca, NY, United States; ^4^ Department of Pediatrics, University of Colorado Anschutz Medical Campus, Aurora, CO, United States; ^5^ Department of Biostatistics and Informatics, Colorado School of Public Health, Aurora, CO, United States; ^6^ Center for Women’s Health Research, University of Colorado Anschutz Medical Campus, Aurora, CO, United States

**Keywords:** POCS, combined oral contraceptives, microbial diversity, adolescent, metabolomics, obesity

## Abstract

**Background:** The gut microbiome is altered in obese adolescents with polycystic ovary syndrome (PCOS), and is associated with free testosterone, metabolic markers, and insulin resistance. Combined oral contraceptives (OCP) are a primary treatment for PCOS and decrease testosterone, but the effect on the serum metabolome or gut microbiome in obese adolescents with PCOS is unknown.

**Objective:** Contrast gut microbiome profiles, targeted serum metabolomics, hormone levels, and metabolic measures in adolescents with PCOS and obesity with and without OCP treatment.

**Methods:** Adolescent girls with obesity and PCOS underwent stool and fasting blood collection and MRI for hepatic fat fraction. Fecal bacteria were profiled by high-throughput 16S rRNA gene sequencing and fasting serum metabolomics performed with high resolution mass spectrometry. Groups were contrasted using t-tests and principle least squares discrimination analysis (PLS-DA). Associations between bacterial taxa, baseline metabolic measures, hormone levels and the metabolome were conducted using Spearman analysis. Analyses were adjusted for false discovery rate.

**Results:** 29 adolescents with obesity [Untreated N = 21, 16 ± 1.2 years, BMI%ile 36.5 ± 3.0; OCP N = 8, 15.5 ± 0.9 years, BMI%ile 32.5 ± 3.9] participated. Of the untreated adolescents, N = 14 contributed serum for metabolomic analysis. Participants on OCP therapy had lower free testosterone and free androgen index (*p* < 0.001) and higher sex hormone binding globulin. There was no difference in measures of fasting glucose, insulin, lipids or HOMA-IR between groups. PLS-DA of serum metabolomics showed discrimination between the groups, secondary amino acid changes. Untreated and OCP had similar stool microbiome α-diversity based on evenness (*p* = 0.28), richness (*p* = 0.39), and Shannon diversity (*p* = 0.24) and global microbial composition (β-diversity, *p* = 0.56). There were no differences in % relative abundance at any level. Bacterial α-diversity was negatively associated with serum long chain fatty acids and branched chain amino acids. A higher %relative abundance of family Ruminococcaceae was significantly associated with serum bile acids and HOMA-IR.

**Conclusion:** Despite hormone and serum amino acid differences, girls treated with OCP had similar metabolic and gut microbiome profiles compared to the untreated PCOS group. The association between bacterial α-diversity, Ruminococcaceae, clinical markers and long chain fatty acids suggests a potential role of the gut microbiome in the pathogenesis of the metabolic comorbidities in PCOS.

## Introduction

Polycystic ovary syndrome (PCOS) is a common endocrine disorder which manifests in adolescence, affecting up to 18% of adolescent females. (Teede et al., 2018) The diagnosis of POCS in adolescents is based on the presence of menstrual irregularity for gynecological age and clinical (i.e., hirsutism or severe acne) or biochemical hyperandrogenism (Teede et al., 2018; Peña et al., 2020) Apart from the reproductive dysfunction of PCOS, several metabolic comorbidities are thought to either exacerbate or drive the development of PCOS, including obesity, insulin resistance, and non-alcoholic fatty liver disease (NAFLD), albeit mechanisms have not been clearly delineated. Metabolic co-morbidities develop early; adolescents with PCOS and obesity have an 18-fold higher incidence of developing type 2 diabetes (Hudnut-Beumler et al., 2021), a prevalence of NAFLD of 40–60%% (Carreau et al., 2019; Andrisse et al., 2021). Understanding the unique etiologies of PCOS which manifest in adolescence and their respective biomarkers are needed for early, effective interventions to prevent or mitigate such deleterious health outcomes.

There is biological plausibility that the interaction between the host and the gut microbiome may drive or exacerbate the reproductive and cardiometabolic disturbances associated with PCOS. Alterations in α-and β-diversity have been reported in adolescents and women with PCOS, albeit with some disagreement in literature. (Rizk and Thackray, 2021) One study demonstrated a causal role for gut microbial disturbances (dysbiosis) driving hyperandrogenism in a mouse model. (Qi et al., 2019) Contrary to a gut-microbiome originated hypothesis, rodent models of hyperandrogenism have also provided evidence that hyperandrogenic models (letrozole, DHT, or DHEA) of PCOS drive changes in gut microbial composition as defined by α- and β-diversity indices. (Rizk and Thackray, 2021) Dysbiosis is also closely tied to the metabolic comorbidities of PCOS. (Rizk and Thackray, 2021) In example, we have previously shown differences in the composition of the gut microbiome between adolescent females with obesity and PCOS compared to those with only obesity, however no differences in gut microbiome composition have been reported between non-obese groups. (Jobira et al., 2020; Mammadova et al., 2021) In summary, although directionality and role of obesity is unclear, collectively these data suggest that dysbiosis is associated with the metabolic and/or reproductive phenotype of PCOS.

Combined oral contraceptives (OCP) are the recommended first-line pharmacological treatments for PCOS in adolescents to improve hyperandrogenism and normalize menstrual cycles. (Peña et al., 2020) Although OCP treatment resolves reproductive phenotype of PCOS, they may also exacerbate the metabolic phenotype of PCOS, notably insulin resistance. (Eyupoglu et al., 2019) Recent studies also suggest exogenous hormone treatment of PCOS may involve gut microbiome-host interactions. Shifts in the gut microbial communities occur in girls during puberty aligning more closely to adult microbiome profiles, particularly in the relative distribution of estrogen-metabolizing microbial communities (Korpela et al., 2021). Inverse shifts in the relative abundance of gut microbial communities have also been demonstrated in women post-menopause, which lends credence to the notion that sex-hormones may underlie shifts in the gut microbiome. These studies suggest that exogenous hormone therapy for PCOS may relate to gut-microbiome mediated changes in the metabolome. However, data are scarce and concurrent microbiome-metabolome data are lacking. Further, there is limited data to date on the effect of OCP in girls with PCOS and obesity. To address these knowledge gaps, the objective of this study was to contrast the gut microbiome with hormonal, glucose, and metabolomic measures among age- and BMI-matched adolescent girls with obesity and PCOS in those treated with and without OCP.

## Methods

### Design

We performed a secondary analysis by using participants from three separate cross-sectional studies. Cases (oral contraceptive use) and controls (no treatment for PCOS) were included.

### Participants

A total of 29 participants were included from 3 separate cross-sectional studies (APPLE NCT02157974, N = 11; PLUM NCT03041129, N = 16; and MISS NCT03120871, N = 2). The stringent National Institute of Health criteria with adolescent adaptation were used to diagnose PCOS: oligomenorrhea defined as <8 menses a year, clinical or biochemical signs of hyperandrogenism and at least 2 years post menarche (Legro et al., 2013). Eight girls were clinically treated with combined oral contraceptives and had been on therapy for at least 6 months (median time 8 months, range 6–24 months), and 21 girls were in the control group and did not receive hormone treatment. Participants were recruited from the pediatric endocrinology and lifestyle medicine outpatient clinics at the Children’s Hospital Colorado. Inclusion criteria were female sex, age 12–20 years, overweight/obesity (BMI >90%ile), Tanner stage 5, post-menarchal status, and sedentary status (<3 h of regular exercise/week; validated with a 3 days activity recall). Exclusion criteria were hypertension, diabetes, medication affecting insulin sensitivity, and antibiotics within 1 month. Information regarding socioeconomic status was not obtained.

### Study Approval

The study protocols were approved by the University of Colorado Anschutz Medical Campus Institutional Review Board and the Children’s Hospital of Colorado Scientific Advisory Review Committee. Informed consent was obtained from all participants and parental consent and participant assent from all participants <18 years old.

### Physical Activity

A 3 days pediatric activity recall (3DPAR) questionnaire was completed with staff assistance from all participants. Participants also wore an accelerometer for 7 days prior to study, with activity scored as sedentary, light, moderate or vigorous.

### Dietary Intake

A diet interview by study staff was completed using a SEARCH food frequency questionnaire (FFQ) to assess macronutrient pattern. FFQ was modified to incorporate and represent common food choices among ethnically and regionally diverse girls age 10–19 years (Mayer-Davis et al., 2006).

### Laboratory Measurements

Anthropomorphic, fasting glucose, sex hormone concentrations, inflammatory markers, lipid, and fasting plasma metabolomic profiles were measured. Two untreated participants from the MISS protocol did not have measurement of sex hormone, AST, HbA1c, C-peptide, complete blood count (platelet and white blood cell), highly sensitive C-reactive protein (hs-CRP), adiponectin, and metabolomics. Analyses were performed by standard methods at the University of Colorado Anschutz Research core laboratory or the Children’s Hospital Colorado clinical laboratory except where noted. Glucose was measured by a Statstrip^©^ hospital grade glucometer (Nova Biomedical, Waltham, MA). Serum insulin and adiponectin were analyzed with RIA (Millipore, Billerica, MA). HbA1c was measured by DCCT-calibrated ion-exchange HPLC (Bio-Rad Laboratories, Hercules, Calif). Alanine aminotransferase (ALT) and aspartame aminotransferase (AST) was measured by multipoint rate with P-5-P method (Vitros® 5600, Ortho Clinical Diagnostics, Raritan, NJ); total cholesterol, high density lipoprotein (HDL) cholesterol, and triglyceride assays were performed enzymatically on a Hitachi 917 autoanalyzer (Boehringer Mannheim Diagnostics, Indianapolis, IN). Low density lipoprotein (LDL) cholesterol levels were calculated by the Friedewald equation; hs-CRP was measured *via* immunoturbidimetric assay (Beckman Coulter, Brea, CA), C-peptide via chemiluminescent immunoassay (DiaSorin, Stillwater, MN), and estradiol via chemiluminescent immunoassay (Beckman Coulter, Brea, CA). Total testosterone was measured by high-pressure liquid chromatography/tandem mass spectrometry, free testosterone *via* equilibrium dialysis and SHBG *via* chemiluminescent immunoassay, all by Esoterix laboratories (Calbassas Hills, CA). DEXA was used to measure percent body fat and lean mass as previously described. (Cree-Green et al., 2016)

### Fecal Collection and Microbiome Analysis

Stool samples were collected at home the day prior to blood sampling using stool collection tubes and frozen in the participant freezer. Upon return to study staff, samples were stored at −80°C until further processing. Bacterial profiles were determined by broad-range analysis of 16S rRNA gene sequences following our previously described methods. (Jobira et al., 2020; Jobira et al., 2021) In brief, DNA was extracted from 50–100 mg of stool using the PowerFecal DNA isolation kit (QIAamp Powerfecal DNA kit (Qiagen INC, Hilden, Germany). Broad-range PCR amplicons were generated using barcoded primers that target the V3V4 variable region of the 16S rRNA gene: primers 338F (5′ ACT​CCT​ACG​GGA​GGC​AGC​AG) and 806R (5′ GGACTACHVGGGTWTCTAAT). PCR products were normalized using a SequalPrep™ kit (Invitrogen, Carlsbad, CA), pooled, and quantified by Qubit Fluorometer 2.0 (Invitrogen, Carlsbad, CA)*.* Illumina paired-end sequencing was performed on the Ilumina MiSeq platform with version v2.3.0.8 of the MiSeq Control Software and version v2.3.32 of MiSeq Reporter, using a 600-cycle version 3 reagent kit. Illumina MiSeq paired-end reads were aligned to a *Homo sapiens* reference genome (UCSC Hg19) with bowtie2 and matching sequences discarded. (Langmead and Salzberg, 2012) Remaining paired-end sequences were demultiplexed and assembled using phrap. (Ewing et al., 1998; Ewing and Green, 1998) Pairs that did not assemble were discarded. Assembled sequences were trimmed over a moving window of 5 nucleotides until average quality met or exceeded 20. Trimmed sequences with more than 1 ambiguity or shorter than 150 nucleotides were discarded. Potential chimeras identified with Uchime (usearch6.0.203_i86linux32) (Edgar et al., 2011) using the Schloss Silva reference sequences (Schloss and Westcott, 2011) were removed from subsequent analyses. Assembled sequences were aligned and classified with SINA (1.3.0-r23838) (Pruesse et al., 2012) using the bacterial sequences in Silva 115NR99 as reference configured to yield the Silva taxonomy. (Pruesse et al., 2012) Taxonomic assignments used SINA’s default parameters for lowest common ancestor naming. Operational taxonomic units (OTUs) were produced by binning sequences with identical taxonomic assignments. Between 37,917 and 195,588 sequence reads were generated per sample and Good’s coverage was >99.0% for all samples.

### Metabolomics Analyses

Serum metabolomics was performed on all of the OCP treatment group and 14 of the control group. Targeted plasma metabolomics analysis was performed at the University of Colorado Metabolomics Shared Resource. Plasma samples were drawn in EDTA tubes, with the plasma separated and frozen immediately at -80°C. Liquid Chromatography-Mass Spectrometry was used for chromatographic separation and mass spectrometry was used for mass detection. Detailed methods have been published previously. (Nemkov et al., 2019; D'Alessandro et al., 2021) Briefly, a volume of 20 μl of plasma aliquots was extracted 1:25 in ice cold extraction solution (methanol:acetonitrile:water5:3:2 v/v/v). Samples were vortexed and insoluble material pelleted, as described. Analyses were performed using a Vanquish UHPLC coupled online to a QExactive mass spectrometer (Thermo Fisher, Bremen, Germany). Samples were analyzed using a 5 and 17 min gradient, as described for analysis of hydrophilic and hydrophobic metabolites (Reisz et al., 2019), respectively. Identification was made comparing masses and retention times from samples to an inhouse library of over 5,000 compounds. (Nemkov et al., 2017) Technical variability was assessed by determination of coefficients of variations (<20%) for metabolite measurements in standard mixes run at intervals of 10 samples. While data was acquired in an untargeted fashion in the range 60–1,500 m/z, stable isotope-labeled internal standards were used for targeted quantitation analysis were selected based on known biologic relationships with insulin resistance and metabolic disease and included the following: Bile acids: taurochenodeoxycholate, tauroursodeoxycholic acid, taurolithocholate, cholic acid, glycocholate, glycochenodeoxycholate, docosahexaenoic acid, eicosapentaenoic acid, and sphingosine 1-phosphate; Branched chain amino acids: alanine, arginine, asparagine, cystine, glutamate, histidine, lysine, methionine, phenylalanine, proline, serine, threonine, tyrosine, valine, isoleucine, leucine, oxoproline, glycine, and spermidine; Fatty acids: arachidonic acid, di-homo-g-linolenic acid, pentaenoic acid, ursodeoxycholic acid, chenodeoxycholic acid, deoxycholic acid, taurodeoxycholate, and taurocholate; Carnitine metabolites: L-carnitine, acetyl-carnitine, propionyl-carnitine, butanoyl-l-carnitine, isovalery-carnitie, L-octanoyl-carnitine, O-tetradecanoyl-L-carnitine, and L-palmitoyl-carnitine.

### Calculations

Insulin resistance and β-cell function were estimated using the Homeostatic Model Assessment (HOMA) model using the formulae: HOMA-IR = (FG * FI)/(405), where FI = fasting insulin µU/mL, FG = fasting glucose (mg/dl).

### Statistical Analysis

Microbiome analysis: Data analyses were performed using R version 3.5.2. The %RA of each taxon was calculated as the number of 16S rRNA sequences of a given taxon divided by the total number of 16S rRNA sequences in a patient’s sample. Differences in overall microbiome composition (β-diversity) between subsets were assessed by a non-parametric, permutation-based multivariate analysis of variance (PERMANOVA with 10,000 replicate resamplings) using Morisita-Horn dissimilarities. Shannon diversity, Shannon evenness, and richness (Sobs) (measures of α-diversity) were calculated using rarefaction and compared across groups using linear models adjusting for batch effects.

Group analysis: Clinical data were evaluated for distribution, and continuous variables between groups compared with students t-tests or Wilcoxon rank sum tests, as appropriate, with a non-adjusted *p*-value of 0.05 considered significant. Categorical variables were analyzed via chi-square tests. Comparisons of %RA across groups were performed using Wilcoxon rank sum (2 groups) tests since batch effects were not significant in any of the individual phylum, family, or genus comparisons, with the *p*-value adjusted for multiple comparisons using the false discovery rate (FDR) method. Partial least-square discriminant analysis (PLS-DA) was performed to contrast the targeted metabolomics between OCP treated and the control group and was adjusted for FDR. Data for this analysis were mean centered and auto scaled, but not log transformed. Spearman’s unadjusted correlations were used to determine the relationship between the gut microbiome (%RA of bacterial taxa at phylum and family, evenness, richness, and diversity) and serum measurements (metabolomic determined concentrations, metabolic, and hormone level variables). Statistics were performed with Sigmastat (version 12), Prism (version 9.3.1) and MetaboAnalyst (5.0).

## Results

### Clinical Characteristic

29 girls who completed the baseline assessment and provided stool sample were included in the final analyses, 8 OCP treated group and 21 of control group. Metabolomic data was available on 8 of the OCP treated and 14 of the control group. Participant demographic, physical characteristics and laboratory measurements are summarized in Table 1. The groups had similar age (*p* = 0.82). Girls with OCP treatment had significantly lower hirsutism (*p* = 0.01). Physical characteristics including acne (*p* = 0.78), BMI (*p* = 0.45), waist-to-hip ratio (*p* = 0.12) were similar. Total body fat (*p* = 0.56) and lean mass (*p* = 0.54) by DEXA were similar. Systolic (*p* = 0.91) and diastolic (*p* = 0.99) blood pressure were similar across groups. There were no differences in diet intake including carbohydrates (*p* = 0.37), protein (*p* = 0.59), and fat (*p* = 0.37) percentages. Activity based on recall survey (*p* = 0.34) and accelerometer data was similar between the groups.

**TABLE 1:** Participant demographic, physical characteristics and laboratory measurements comparing girls with obesity and PCOS with (N = 8) and without (N = 21) oral contraceptive treatment. PCOS untreated had significantly higher hirsutism score, free testosterone, and free androgen index and lower SHBG and platelets. All other variables were similar between groups.

Variables	PCOS, untreated (N = 21)	PCOS OCP-treated (N = 8)	*p*-value
Demographics and History			
Age (years)	16 (15.5, 17)	15.5 (15, 17)	0.82
Race (Caucasian, Black, Asian %)	76, 14, 10	100, 0, 0	0.32
Ethnicity (Hispanic, Non-Hispanic)	57, 43	50, 50	0.73
Menarche Age (years)	11.5 ± 1.3	11.9 + 1.4	0.54
Physical Characteristics			
Acne	2 (1, 2)	1 (0.3, 1.8)	0.78
Hirsutism (FGS score)	**8.9** ± **5.4**	**2.5** ± **2.3**	0.01
Waist:hip ratio	0.91 (0.86, 0.95)	0.92 (0.84, 0.98)	0.12
BMI (kg/m2)	36.5 (33.0, 39.5)	32.5 (31.1,39.8)	0.45
BMI (%ile)	98.8 (97.5, 99.1)	97.6 (96.6, 99.1)	0.29
BMI Z-score	2.1 (2.0, 2.4)	2.0 (1.8, 2.4)	0.29
Systolic BP (mmHg)	120 (155,125)	117 (112, 125)	0.91
Diastolic BP (mmHg)	71 (68, 77)	71 (67, 76)	0.99
% body fat by DEXA	45.4 ± 3.9	46.4 ± 4.0	0.56
% lean mass by DEXA	52.0 ± 3.8	51.1 ± 3.8	0.54
7 Day Dietary Intake Recall			
Fat intake (%)	41 (37, 48)	41 (36, 43)	0.37
Protein intake (%)	16 (14, 18)	15 (13, 16)	0.59
Carbohydrate intake (%)	43 (35, 49)	44 (41, 49)	0.37
Physical Activity			
Activity from recall survey (METS)	55.3 ± 8.7	51.6 ± 10.2	0.34
Accelerometer % Sedentary	51 ± 14	55 ± 7	0.47
Accelerometer % Light	36 ± 10	35 ± 4	0.75
Accelerometer % Moderate or Vigorous	7 ± 7	6 ± 7	0.87
Laboratory Measurements			
Free testosterone (ng/dl)	**9.2 (7.8, 13)**	**2.3 (1.7, 3.7)**	**<0.01**
Total testosterone (ng/dl)	41 (33, 51)	30 (24, 49)	0.288
SHBG (mmol/L)	**18.6 (12, 22)**	**100 (64, 124)**	**<0.01**
Free Androgen Index	**9.0** ± **3.2**	**1.3** ± **0.5**	**<0.01**
Estradiol (pg/ml)	53 (45, 80)	35 (28, 49)	0.09
AST(IU/mL)	50 (38, 79)	32 (30, 48)	0.14
ALT (IU/ml)	35 (30, 46)	37 (31, 41)	0.54
WBC (10^9^ cells/L)	8.5 ± 1.9	8.5 ± 2.7	0.99
hs-CRP (mg/dl)	2.4 (1.1, 7.1)	9.5 (2.9, 12.7)	0.09
Adiponectin (ng/ml)	6.4 (4.9, 10.4)	5.7 (5.1, 8.0)	0.57
Platelets (10^8^ cells/L)	**310 (283, 334)**	**333 (323, 354)**	**0.01**
Cholesterol (mg/dl)	140 (133, 175)	162 (137, 180)	0.41
HDL (mg/dl)	35 (29, 44)	41 (38, 46)	0.19
LDL (mg/dl)	103 (91, 127)	121 (100, 137)	0.35
Triglycerides (mg/dl)	112 (105, 150)	144 (93, 195)	0.63
HbA1c (%)	5.6 (5.3, 5.8)	5.5 (5.3, 5.7)	0.85
Fasting glucose (mg/dl)	86 (80, 92)	88 (83, 93)	0.57
Fasting insulin (μU/mL)	23 (19, 42)	26 (18, 36)	0.68
Fasting C-peptide (ng/ml)	2.3 (1.8, 3.4)	2.6 (2.0, 3.1)	0.99
2 h glucose (mg/dl)	127 ± 26	151 ± 38	0.08
2 h insulin (μU/mL)	185 (86, 649)	167 (107, 314)	0.65
HOMA-IR	5.1 (3.3, 7.9)	5.0 (4.6, 11.5)	0.62
Matsuda Index	1.4 (0.8, 1.9)	1.6 (0.9, 2.5)	0.89

Values are mean ± standard error of the mean, or median (25%, 75%). BMI, body mass index; METS, metabolic equivalents; SHBG, sex hormone binding globulin; HDL, high density lipoprotein; LDL, low density lipoprotein; HbA1c, hemoglobin A1c. AST, aspartate transferase; ALT, alanine transferase; hsCRP, highly sensitive c-reactive protein. HOMA-IR, homeostatic assessment insulin resistance. Significant values highlighted in bold.

The OCP treated group had significantly lower free testosterone (*p* < 0.001), free androgen index (*p* < 0.001) and higher SHBG (*p* < 0.001). Total testosterone and estradiol were similar in both groups. There were no group differences in triglycerides, cholesterol, HDL-C, LDL-C, fasting or 2 h glucose and insulin, HbA1c, C-peptide or adiponectin. Calculated measures of insulin resistance, HOMA-IR and Matsuda score were not different between groups. The OCP treated group had significantly higher platelets (*p* = 0.01) compared to the control group; however, all other markers of inflammation including WBC (*p* = 0.99) and hs-CRP (*p* = 0.09) were similar between groups. There were no differences in AST and ALT between groups.

### Gut Microbiome Diversity and Composition Across Groups

**FIGURE 1:**
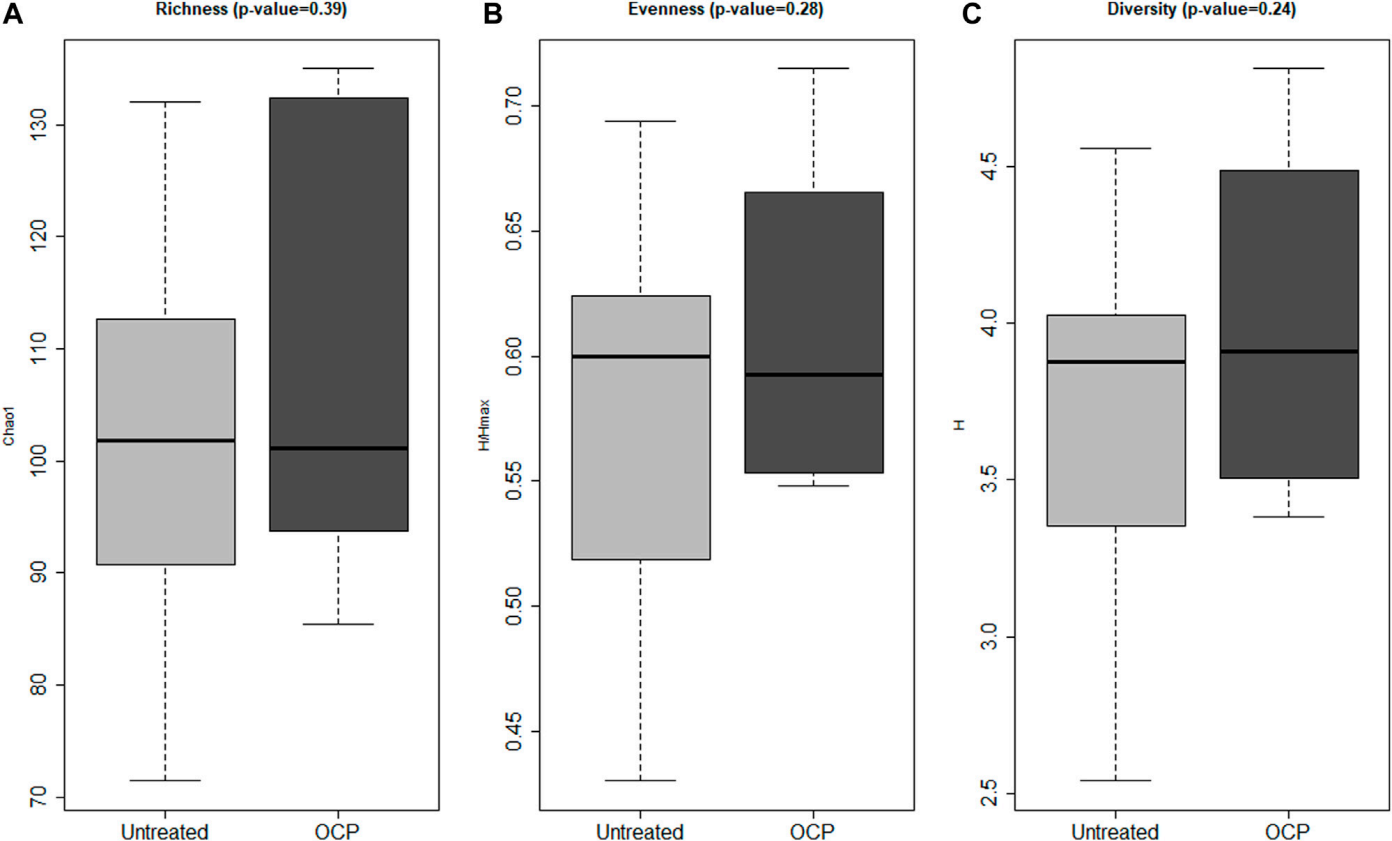
Bacterial α-Diversity. Measures of α-biodiversity including: **(A)** Richness (Chao1) **(B)** Evenness (Shannon H/Hmax), **(C)** Shannon Diversity (Shannon H). There were no statistical differences in α-diversity between groups. Shannon diversity, Shannon evenness, and richness (Sobs) (measures of α-diversity) were calculated using rarefaction and compared across groups using linear models adjusting for batch effects. Girls with obesity and PCOS with (N = 8) and without (N = 14) oral contraceptive treatment were included.

Bacterial 16S rRNA gene profiling was completed for all samples; both groups had adequate depth of sequencing coverage (Good’s coverage of >99.0% for all samples) indicating comparable and representative samples. Measures of α-diversity including richness (*p* = 0.39), evenness (*p* = 0.28), and complexity (*p* = 0.24) were similar across groups (Figure 1). There was no difference in β-diversity (*p* = 0.56) across groups at the genus level as assessed by PERMANOVA (data not shown).

**FIGURE 2:**
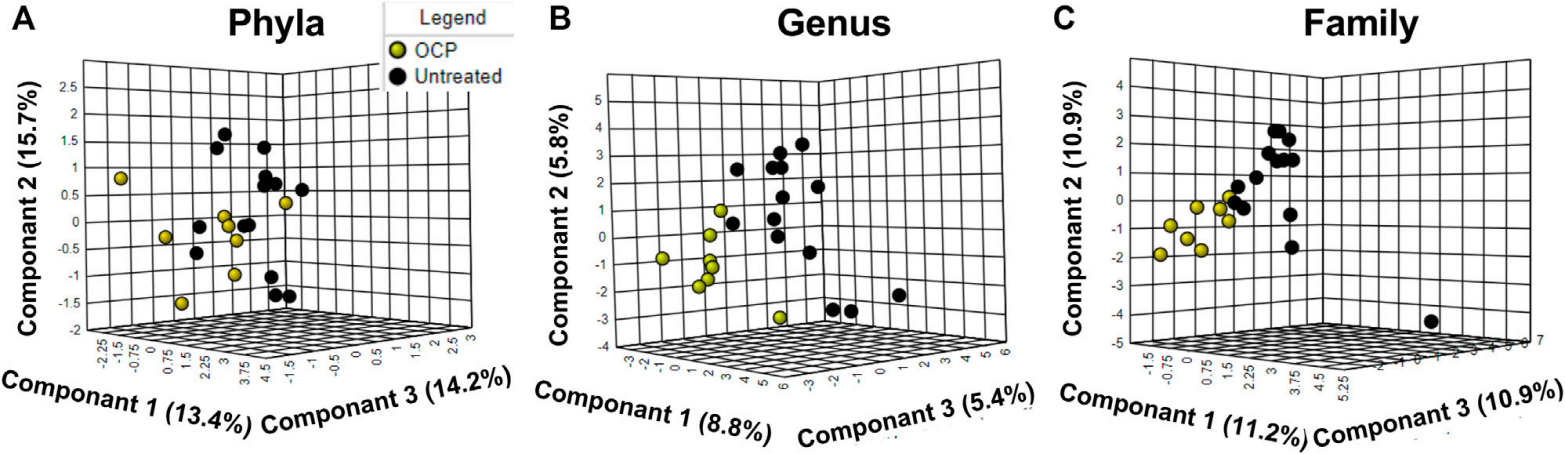
**(A)** Phyla; **(B)** Genus; **(C)** Family Microbiome differences between OCP and untreated. Partial Least Square-Discriminant Analysis (PLS-DA) of bacterial % relative abundance between groups at the **(A)** Phyla **(B)** Genus and **(C)** Family level. Only taxa with > 1% RA are included. For Phyla, principle component 2 explained 15.7% of the total variance; for genus, Principle component 1 explained 8.8% of total variance; for family, Principle Component 1 explained 11.2% of the variance. Girls with obesity and PCOS with (N = 8) and without (N = 14) oral contraceptive treatment were included.

PLS-DA across phyla, family, and genus are depicted in Figure 2 with clear overlap noted in all figures. Further, Variable Importance in Projection (VIP) analysis (not shown) confirmed no differences between the groups. For Phyla, principle component 2 explained 15.7% of the total variance, Principle component 1 explained 8.8% of the total variance for genus and Principle Component 1 explained 11.2% of the total variance for family. As expected for human fecal microbiota, the most predominant phyla were Firmicutes, Bacteroidetes, Actinobacteria, and Proteobacteria*,* respectively (Jobira et al., 2020; Jobira et al., 2021) However, no differences between groups at the phylum or family level were detected (data not shown). However, at the genus level, the %RA of *Pseudobutyrivibrio* was significantly higher in the OCP group (*p* = 0.045); no other differences at the genus level were detected (data not shown)

### Metabolomic Comparison Across Groups

**FIGURE 3:**
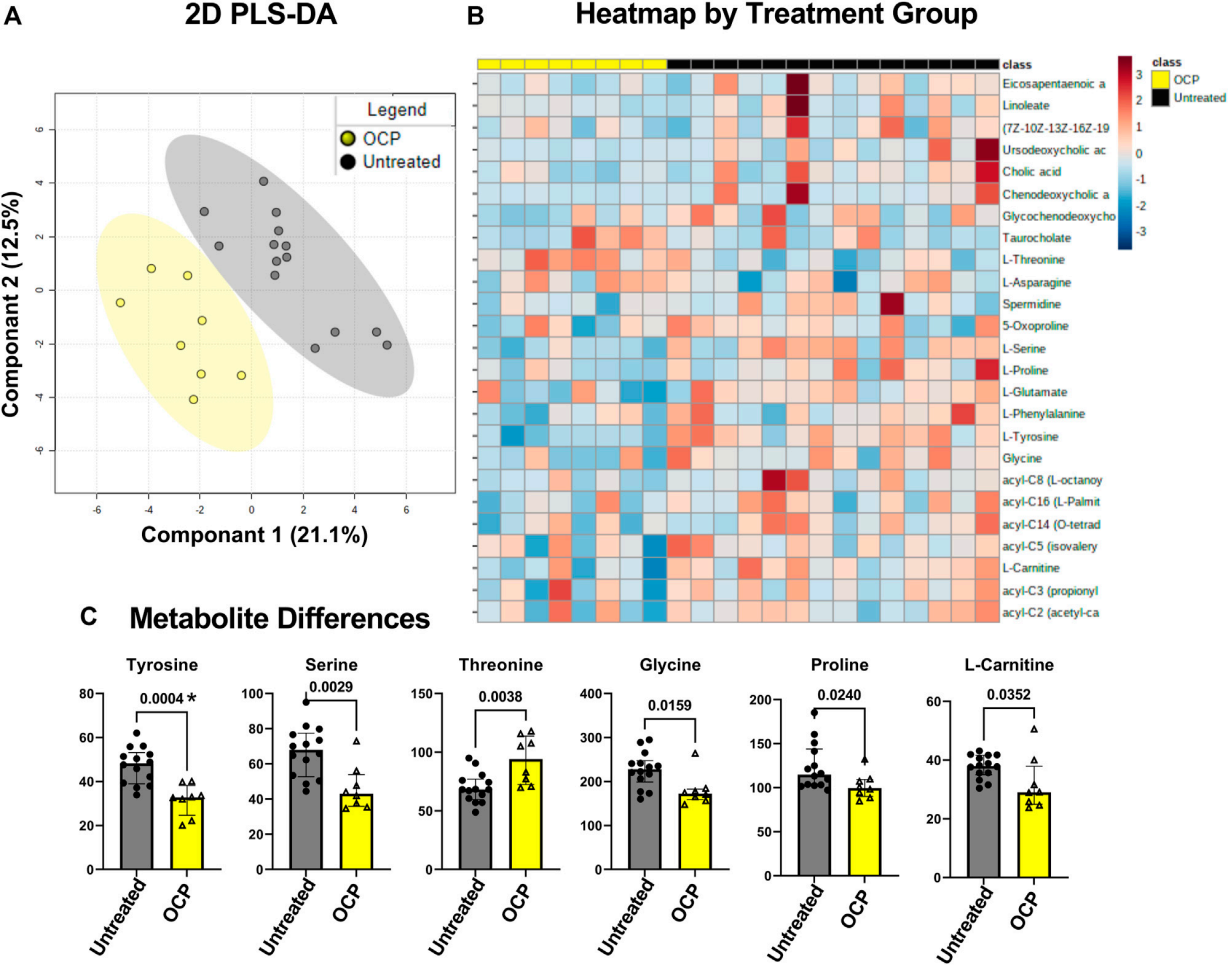
Partial least-square discriminant analysis (PLS-DA) of target Metabolomics by group. **(A)** PLS-DA of targeted metabolomics including free fatty acids, bile acids, and branched chain amino acids was different between groups, PLS-DA showed discrimination between the two groups across principal component 1, which explained 21.1% of the total metabolic variance across samples **(B)** Heatmap of top 25 metabolites **(C)** 6 serum metabolites were different between the groups with unadjusted *p*-value. Only Tyrosine marked *, remained so with False Discovery Rate adjustment for multiple comparisons. Girls with obesity and PCOS with (N = 8) and without (N = 14) oral contraceptive treatment were included.

PLS-DA on 22 participants, girls with obesity and PCOS with (N = 8) and without (N = 14) OCP treatment, showed discrimination between the two groups across principal component 1, which explained 21.1% of the total metabolic variance across samples (Figure 3A). Differences per individual are depicted in the heatmap of the top 25 compounds (Figure 3B). These differences unadjusted for multiple comparisons were driven by a significantly lower concentration of amino acid L-threonine, and lower concentrations of L-tyrosine, L-serine, L-glycine, and L-proline, and free L-carnitine in the OCP group compared to the untreated group (Figure 3C). However, only Tyrosine remained significantly different following FDR adjustment for *p*-values.

### Correlation Between Bacterial Taxa and α-Diversity Indices With Metabolic, Hormonal and Metabolomic Markers

Exploratory unadjusted analysis of serum clinical variables, hormonal, and metabolomic measurements were evaluated for correlations between α-diversity indices, phyla, and family level bacterial taxa. Refer to Table 2 for the correlation result. Free testosterone was significantly correlated with α-diversity including bacterial evenness (R = −0.58, *p* = 0.005), bacterial diversity (R = −0.55, *p* = 0.008). HOMA-IR was significantly correlated with family Ruminococcaceae (R = −0.43, *p* = 0.043). All other clinical variables were not statistically significant. We included variables with *p*-values ≤0.06 and are noted on Table 2 as not significant (NS)

**TABLE 2:** Variables from Table 1 were run for unadjusted correlation with α-diversity indices and phyla and family bacterial taxa. All participants including girls with obesity and PCOS with (N = 8) and without (N = 21) oral contraceptive treatment were included. Free testosterone, HOMA-IR, and fatty acids, bile acids, branched chain amino acids were significantly correlated with Evenness, Shannon diversity and Ruminococcaceae. When significant, results are shown with R value, (*p*-value).**NS = not significant, *p*-values >0.06.

Variables* (targeted metabolomics, hormonal and glucose measures)	Evenness (R, *p*-value***)	Shannon diversity (R, *p*-value***)		Ruminococcaceae (R, *p*-value***)
Free testosterone	−0.58 (0.005)	−0.55 (0.008)	
HOMA-IR				−0.43 (0.043)
Linoleate	−0.51 (0.014)	−0.53 (0.010)	
7Z-10Z-13Z-16Z-19Z (Pentaenoic acid)	−0.49 (0.020)	−0.51 (0.015)		NS
acyl-C8 L-octanoyl	−0.45 (0.035)	−0.47 (0.026)		NS
Dihomo-g-linolenic	−0.46 (0.029)	−0.50 (0.018)		−0.43 (0.044)
Ursodeoxycholic acid (UDCA)	NS	NS		−0.48 (0.022)
Taurocholate (TCA)	NS	NS		0.45 (0.036)
Taurolithocholate (TLCA)	NS	NS		0.44 (0.040)
Taurodeoxycholate (TDCA)	NS	NS		0.60 (0.003)
Alanine	−0.53 (0.011)	−0.55 (0.008)		−0.52 (0.012)
L-Valine	−0.49 (0.021)	−0.50 (0.016)		NS
L-Isoleucine	−0.41 (0.061)	−0.40 (0.060)		NS
L-Leucine	−0.43 (0.042)	−0.43 (0.043)		NS
Glycine	NS	NS		−0.45 (0.033)
Acyl-C8 L-octanoyl	−0.45 (0.035)	−0.47 (0.026)		NS

*Variables from Table 1 were run for correlation with α-diversity indices and phyla and family bacterial taxa (N = 22). When significant, results are shown with R value, (*p*-value); NS, not significant, *p*-values >0.06.; ***p-values are unadjusted for multiple comparison.

Several fatty acids such as Linoleate were significantly correlated with α-diversity indices measuring evenness (R = −0.51, *p* = 0.014) and Shannon diversity (R = −0.53, *p* = 0.010); Pentaenoic acid was significantly correlated with evenness (R = −0.49, *p* = 0.020) and Shannon diversity (R = −0.51, *p* = 0.015); Dihomo-g-linolenic was significantly correlated with evenness (R = −0.46, *p* = 0.029), Shannon diversity (R = −0.50, *p* = 0.018); Acyl-C8-L-octanoyl was significantly associated with evenness (R = -0.45, *p* = 0.035) and Shannon diversity (R = -0.47, *p* = 0.026).

Several metabolites were significantly associated with family Ruminococcaceae including a polyunsaturated fatty acid, Dihomo-g-linolenic (R = −0.43, *p* = 0.044), several bile acids (Ursodeoxycholic acid (R = −0.48, *p* = 0.022), Taurocholate (R = 0.45, *p* = 0.036), Taurolithocholate (R = 0.44, *p* = 0.040), Taurodeoxycholate (R = 0.60, *p* = 0.003)), and the amino acid Glycine (R = −0.45, *p* = 0.033). The following branched chain amino acids were significantly associated with α-diversity: Alanine with evenness (R = −0.53, *p* = 0.011) and Shannon diversity (R = −0.55, *p* = 0.008), L-Valine with evenness (R = -0.49, *p* = 0.021) and Shannon diversity (R = -0.50, *p* = 0.016), L-isoleucine with evenness (R = -0.41, *p* = 0.061) and Shannon diversity (R = -0.40, *p* = 0.060), and l-leucine with evenness (R = -0.43, *p* = 0.042) and Shannon diversity (R = −0.43, *p* = 0.043).

## Discussion

We sought to contrast the gut microbiome, hormonal measures, and a focused sample of the serum metabolome between adolescent girls with obesity and PCOS who were actively taking OCP versus those naïve to treatment. There was an overall difference in the serum metabolome, with predominate factors including amino acids and creatine. Differences in the gut microbiome were not observed at the community or aggregate level. Further, we did not detect group differences in α- or β-diversity between the OCP treated and untreated, indicating that no major shifts in the overall microbiome profiles occurred with OCP therapy. When all participants were combined, we observed associations between α-diversity indices and free testosterone, long chain fatty acids, and branched chain amino acids alongside significant correlations between the %RA of Ruminococcaceae and conjugated primary and secondary bile acids. These results suggest that treatment with OCP is not associated with differences in the stool microbiome or related lipid or bile acids in the serum metabolome. However, lower circulating L-tyrosine, L-serine, L-glycine, and L-proline, and free L-carnitine and a higher concentration of L-threonine was detected in the OCP group versus the untreated.

Limited evaluation of the gut microbiome has been conducted in adolescents with PCOS who were treated with OCP. A recent study contrasted changes in the gut microbiome in Catalonian adolescents with PCOS without obesity before and after treatment with OCP and combination of spironolactone, pioglitazone, and metformin (SPIOMET). (Garcia-Beltran et al., 2021) No differences in α- or β-diversity were noted after treatment in the OCP group, the group receiving SPIOMET exhibited a reduction in the relative abundance of *Family XI* alongside reductions in hepato-visceral fat. It is possible that changes in the gut microbiome and downstream effects on host physiology may have occurred following SPIOMET, but not OCP use, because of direct actions on the microbiome. OCP use, in contrast, would be expected to first induce changes in serum concentrations of androgens which would then be anticipated to influence gut microbiome composition. In a separate study, adult women with PCOS who underwent a 3-months OCP treatment exhibited reduced circulating gut microbiome metabolite trimethylamine N-oxide (TMAO) which was correlated in a change in free androgen index (FAI) after treatment, suggesting that improvements in hyperandrogenism occur concurrent with reductions in TMAO. (Eyupoglu et al., 2019) Although gut microbiome composition was not evaluated in the latter, these data suggest that OCP may induce shifts in the metabolic activity of the gut microbiome. Whether shifts in hormonal composition were sufficient to induce changes in the metabolic activity of the gut microbiome was not investigated in the present study but it a potential area for future research. Our study, and that of Ibanez et al., stand in contrast to studies in animal models that have documented significant changes in α- and β-diversity of gut microbiota following gonadectomy or administration of exogenous hormones. (Org et al., 2016) It is plausible that the dose of exogenous hormones and duration of OCP exposure in human studies may not have been sufficient to drive detectable differences in α-diversity indices. Alternatively, it could be that the narrow and controlled diet of animal models allows for the detection of change with estrogen therapy, whereas in humans with a varied diet, the effect of OCP is not so great as to overcome the influences of diet.

We found that the serum metabolome in aggregate did differ between groups per PLS-DA analysis. Specifically, differences in the serum concentrations of tyrosine, glycine, serine, threonine and proline—amino acid substrates involved in gluconeogenesis previously identified as markers of obesity (Remesy et al., 1983; Alves et al., 2019) and/or prediabetes. (Chen et al., 2019; Just et al., 2020). Testosterone has been shown to increase protein synthesis and decrease protein clearance via the urea cycle, both of which will alter serum amino acid concentrations (Ferrando et al., 2003; Lam et al., 2017) Our previous work demonstrated that fasting concentrations of tyrosine were higher in those with PCOS and obesity compared to obesity alone, so it is notable that this decreased with OCP treatment. (Cree-Green et al., 2019) However, the remainder of the fasting concentrations of amino acids were not different with PCOS status alone. (Cree-Green et al., 2019) Sex-specific differences in the metabolome between men and women have implicated sex steroids in the regulation of the metabolome, especially amino acids as relates to increased protein synthesis and turnover. (Ruoppolo et al., 2014) Exogenous steroid hormone administration has also been shown to alter the metabolome in women; in healthy women, OCP use (ethinylestradiol-progestin preparation) is associated with differences in amino acid profiles within the metabolome. (Ruoppolo et al., 2014) Because cohorts were intentionally matched for obesity status and race and ethnicity, all factors known to influence the serum metabolome, we interpret these findings to suggest that OCP effects on ameliorating the hyperandrogenism of PCOS are primarily manifest in the direct action of testosterone on amino acid metabolism alone. There are both biological and methodological interpretations of our findings. We have previously demonstrated that adolescents with PCOS and obesity do have alterations in the serum metabolome compared to girls without PCOS. (Cree-Green et al., 2019) We had anticipated that the OCP would change the metabolome towards that seen in the controls. Consistently, the observed changes are known to be related to insulin sensitivity and metabolic disease and altered in PCOS per our previous work. That said, the inter-individual variability of the non-amino acid metabolome in PCOS has been reportedly more variable versus healthy, reference population (Ozegowska et al., 2021). Therefore, it is plausible that changes induced by OCP may not exceed the effect size of the inter-individual variability of the metabolome within PCOS, per se.

We did however find that the relative abundance of *Pseudobutyrivibro* was higher in the treated group compared to the untreated cohort. The genus *Pseudobutyrivibro* stems from the *Lacnospiraceae* family belonging to the phylum *Firmicutes*. A recent study reported an increase in the relative abundance of *Pseudobutyribrio* alongside increases in circulating estradiol and progesterone in female mice following red clover supplementation. (Chen et al., 2021) This is consistent with the %RA of *Pseudobutyrivibro* being greater in adolescents taking OCP. To our knowledge, data on steroidogenic regulation of *Pseudobutyrivibro* are lacking in humans. Therefore, we interpret our data cautiously and report an association without a clear mechanistic relationship. Whether OCP induce selective changes in the relative abundance of species, and the nutritional versus pharmacological relationship between sex steroids and gut microbiome composition is intriguing and worthy of future study.

We also found that a lower relative abundance of Ruminococcacea was associated with more pronounced insulin resistance as judged by HOMA-IR and greater concentrations of taurine-conjugated primary (TCA) and secondary (TLCA, TDCA) bile acids. Because the gut microbiome participates in bile acid deconjugation, these findings are expected. Ruminococcacea produce short chain fatty acids (SCFA), which are derived from microbiome-metabolized starches and are important for gut integrity and immune function. (Liu et al., 2017) Lower relative abundance of Ruminococcacea has been reported in PCOS and was negatively associated with degree of hyperandrogenism defined by total testosterone. (Liu et al., 2017) Furthermore, the relative abundance of Ruminococcacea was associated with castration in male mice, suggesting a direct modulatory effect of androgens on the microbiome, specifically the Ruminococcacea. Others have posited a role of Ruminococcacea in the regulation of bioavailable sex-hormones. (Korpela et al., 2021) These studies suggest that Ruminococcacea is involved in the metabolic and hormonal interplay in obesity and PCOS but it is specific role and contribution in the pathophysiologic mechanism is an area for further investigation.

When participants were considered together, regardless of contraceptive use, we found that α-diversity, notably species evenness and Shannon diversity, were negatively associated with degree of free testosterone, branched chain amino acids, and long chain fatty acids as shown in Table 2. Lower α-diversity has been reported in adolescents and adults with PCOS in some, but not all studies (reviewed in detail elsewhere). (Rizk and Thackray, 2021) We anticipate that the effect of contraceptive use, as discussed earlier, may have been insufficient in dose or duration to overcome any effect of PCOS status.

This study has several strengths, primarily relating to the careful selection and comprehensive phenotype of the participants. The groups are similar in terms of age, BMI and race ethnicity, diet and physical activity, all factors that may affect the microbiome. PCOS status was confirmed using the stringent NIH criteria. This study also had limitations. First, we acknowledge the small sample size, particularly for the complex datasets generated. However, this is a sub-analysis representing a pilot investigation composed of participants from three studies; the present analysis is hypothesis generating and intended to inform future studies on study design, methods, and sample size calculation. Secondly, participants on OCP had selected this therapy, and they may have differed from the untreated group prior to treatment with the OCP.

In conclusion, OCP use in adolescents is not associated with detectable shifts in the gut microbiome, but may reflect downstream metabolic effects of testosterone, especially as relates to serum amino acids. The lack of major gut microbiome and metabolic change with OCP may relate to the minimal improvement seen in clinical metabolic disease in patients with PCOS. It also suggests that elevated androgens alone are not responsible for metabolic disease in PCOS, and further investigation into the comprehensive effect of OCP’s is needed.

## Data Availability

The original contributions presented in the study are publicly available. This data can be found here: https://www.ncbi.nlm.nih.gov/, PRJNA681631.
